# Nitriding an Oxygen-Doped Nanocarbonaceous Sorbent Synthesized via Solution Plasma Process for Improving CO_2_ Adsorption Capacity

**DOI:** 10.3390/nano9121776

**Published:** 2019-12-13

**Authors:** Phuwadej Pornaroontham, Gasidit Panomsuwan, Sangwoo Chae, Nagahiro Saito, Nutthavich Thouchprasitchai, Yuththaphan Phongboonchoo, Sangobtip Pongstabodee

**Affiliations:** 1 Department of Chemical Technology, Faculty of Science, Chulalongkorn University, 254 Phayathai Road, Pathumwan, Bangkok 10330, Thailand; phuwadej.p@gmail.com (P.P.); nutthavich_t@hotmail.com (N.T.); yuththaphan.p@gmail.com (Y.P.); 2 Department of Materials Engineering, Faculty of Engineering, Kasetsart University, Bangkok 10900, Thailand; fenggdp@ku.ac.th; 3 Department of Chemical Systems Engineering, Graduate School of Engineering, Nagoya University, Furo-cho, Chikusa-ku, Nagoya 464-8603, Japan; chae@sp.material.nagoya-u.ac.jp (S.C.); hiro@sp.material.nagoya-u.ac.jp (N.S.); 4 Center of Excellence on Petrochemical and Materials Technology, Chulalongkorn University, 254 Phayathai Road, Pathumwan, Bangkok 10330, Thailand

**Keywords:** carbon dioxide, adsorption, solution plasma process, carbonaceous material

## Abstract

The synthesis of carbon nanoparticles (Cn) and oxygen-doped nanocarbon (OCn) was successfully done through a one-step synthesis by the solution plasma process (SPP). The Cn and OCn were nitrogen-doped by nitridation under an ammonia atmosphere at 800 °C for 2 h to yield NCn and NOCn, respectively, for carbon dioxide (CO_2_) adsorption. The NOCn exhibited the highest specific surface area (~570 m^2^ g^−1^) and highest CO_2_ adsorption capacity (1.63 mmol g^−1^ at 25 °C) among the synthesized samples. The primary nitrogen species on the surface of NOCn were pyridinic-N and pyrrolic-N. The synergistic effect of microporosity and nitrogen functionality on the NOCn surface played an essential role in CO_2_ adsorption enhancement. From the thermodynamic viewpoint, the CO_2_ adsorption on NOCn was physisorption, exothermic, and spontaneous. The NOCn showed a more negative enthalpy of adsorption, indicating its stronger interaction for CO_2_ on the surface, and hence, the higher adsorption capacity. The CO_2_ adsorption on NOCn over the whole pressure range at 25–55 °C best fitted the Toth model, suggesting monolayer adsorption on the heterogeneous surface. In addition, NOCn expressed a higher selective CO_2_ adsorption than Cn and so was a good candidate for multicycle adsorption.

## 1. Introduction

One potential warning sign from nature to human beings is the increasing occurrence of severe disasters. Some parts of the earth face a cruel drought, while others have flash floods, and this threatens ecosystems and seasonal variations. One major proposed cause of these changes is the excess carbon dioxide (CO_2_) emissions from anthropogenic activities to the atmosphere. The CO_2_ emissions also exceed the net CO_2_ depletion rate by, for example, photosynthesis. Accordingly, international cooperation has led to an almost global declaration to reduce the level of CO_2_ emission into the atmosphere. Some governments encourage afforestation in their countries to soak up CO_2_ from the atmosphere, while a broad focus is to investigate ways to eliminate CO_2_ emissions from gaseous effluent streams, particularly from post-combustion flue gas, before releasing to the atmosphere. In practice, CO_2_ is reacted with an amine solution in the scrubbing unit. However, this method has many drawbacks in terms of the equipment corrosion, high energy consumption for the regeneration of the amine, requirement of a large space for installing the scrubbing-unit [1,2], and loss by evaporation of the environmentally toxic amine solution.

An efficient alternative method for CO_2_ removal is the adsorption of CO_2_ onto a porous solid sorbent, due to its ease of handling and low energy consumption for regeneration. Porous sorbents with amine-functionalization, such as zeolites [3], silicas [4,5], activated carbons [6], and polymers [7], have been investigated for their adsorption performance. It has been found that the pore structure, type of amine, number of active sites, and the operating condition have a significant influence on the CO_2_ adsorption capacity of the sorbent. An amine-functionalized sorbent expresses a high CO_2_ adsorption capacity, since the amine reacts with CO_2_ via a base-acid reaction to form carbamate [8,9], and/or bicarbonate species [1,10]. However, some of the active amine species are lost when operating or regenerating at a high temperature. Consequently, the CO_2_ adsorption capacity drops dramatically. A suitable sorbent should possess not only a high selective CO_2_ adsorption capacity, but also the ability to be regenerated without deactivation. To address this problem, doping a nitrogen atom onto the framework of the carbon-based sorbent has been proposed as a promising choice owing to its high CO_2_ adsorption capacity and weaker binding energy (BE) to CO_2_ in comparison with conventional amine-functionalized sorbents [11,12,13].

The most widely used method to prepare carbon-based sorbents is based on carbonization or pyrolysis of solid carbon materials at a high temperature. Previous studies revealed that the microporous [14,15], and mesoporous structure [16,17] are crucial factors in determining the adsorption capacity of the carbon sorbent. Changing its skeleton to a nanoscale enhances faster adsorption kinetics [18,19]. In general, doping nitrogen atoms onto the carbon matrix via heat treatment in ammonia (NH_3_)-flow atmosphere promotes the CO_2_ adsorption capacity [20,21]. However, a greater amount of nitrogen can be doped on carbon frameworks that have a higher oxygen density on the matrix surface [22]. From these points, it can be noted that at least two steps (carbonization of the carbon material and an increase in the surface oxygen density) are required to prepare an oxygen-doped carbon substrate prior to nitridation. Therefore, to save the cost and energy consumption for synthesizing an oxygen-doped carbonaceous sorbent, a one-step synthesis at room temperature and atmospheric pressure is highly needed.

Recently, the solution plasma process (SPP), a cold or non-equilibrium plasma in the liquid phase [23], has emerged as a powerful method for synthesizing carbon materials with various dopants. The SPP can be performed without external heating and pressurizing. In an SPP reactor, the liquid organic molecules, which the plasma passes through, absorb sufficient plasma energy and are activated to excited molecules and radical species. These activated molecules act as an initiator to induce chemical reactions, leading to the formation of carbon materials. The type of organic solution has an essential role in designing the desired species in the carbon framework. For instance, a mixture of pure organic liquids is applied to synthesize heteroatom-doped carbonaceous materials [23,24]. However, there have been very few reports on using a mixture of non-pure organic solutions to synthesize oxygen-doped carbonaceous sorbents. Generally, metalworking fluid (MWF) is used in workshops for metal-cutting in order to transfer heat at the interface between the tool and workpiece, to lubricate the tool, workpiece, and machine, to inhibit corrosion and remove some fines, chips and swarfs. With respect to other uses of MWF, it would be the first time to apply MWF as one of the precursors for the synthesis of carbonaceous particles via SPP. The MWF used in this work was comprised of >80% hydrocarbon oil and ~20% oxygen-containing cosolvent and additives.

Here, we aimed to investigate the CO_2_ adsorption capacity of solid carbon sorbents synthesized by SPP. Pure benzene was used to synthesize carbonaceous sorbents, while a mixture of benzene and MWF was used to synthesize oxygen-doped carbonaceous sorbents. The role of benzene was to act as a precursor for the skeleton of the carbon matrix synthesis, while the MWF acted as the oxygen source. Another important reason to choose MWF is that it has complete miscibility with benzene. Moreover, we also studied the enhancement of the CO_2_ adsorption capacity via nitriding at 800 °C in an NH_3_ atmosphere. The sorbents were characterized to analyze the morphology, structure, and chemical bonding state. In addition, the adsorption thermodynamic, selective CO_2_ adsorption, cyclic adsorption/regeneration, and isotherm studies were evaluated in order to reveal the CO_2_ adsorption behavior on the sorbents.

## 2. Materials and Methods

### 2.1. Sorbent Preparation

**Figure 1:**
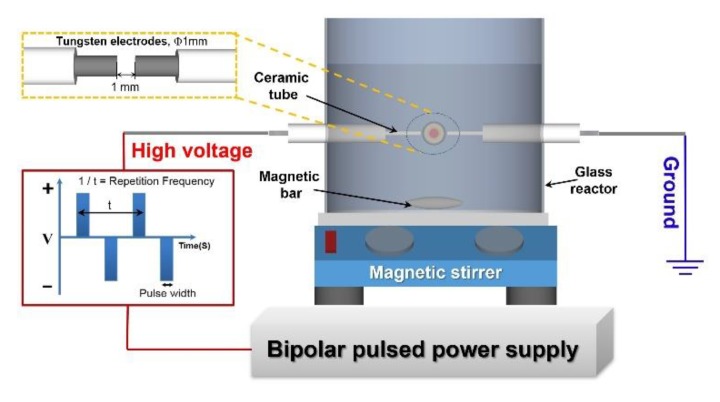
Schematic representation of the SPP.

The SPP equipment is shown schematically in Figure 1. A pair of tungsten electrodes (1 mm diameter) were sandpapered to clean their surface and then insulated with ceramic tubes and fitted to the 100 mL glass reactor vessel using a silicone bung. The gap between the two electrodes was set at 1 mm.

To synthesize the carbonaceous nanoparticles (Cn), 100 mL of benzene (Quality Reagent Chemical; Qrec^TM^, Chonburi, Thailand) was placed into the SPP reactor. After setting the operating parameters to 1.5 kV voltage, 1.0 μs pulse width and 20 kHz applied pulse frequency; and the electrodes were connected to a bipolar pulse power supply to introduce the plasma for 30 min. The pale clear yellow solution became a deep-black suspension, and the tiny black particles were harvested by filtration and washed with hexane (Quality Reagent Chemical; Qrec^TM^) until the washing solution turned colorless. The particles were then washed several times with ethanol (99.9% purity, Quality Reagent Chemical; Qrec^TM^) and oven-dried at 80 °C for 12 h. The resultant particles were kept in a dark bottle and stored in a desiccator. In order to synthesize the oxygen-doped carbonaceous nanoparticles (OCn), 100 mL of 15% (*v*/*v*) MWF (DMSO < 3%, additives: Surfactant; corrosion inhibitor; stabilizer; preservative, DROMUS BA, Thai Houghton 1993 company, Rayong, Thailand) in benzene was then used.

To obtain the nitrogen-oxygen codoped carbonaceous sorbent (NOCn) by the combination of SPP and nitridation, 2.0 g of OCn contained in a quartz boat was placed in a horizontal quartz tubular reactor of 38 mm inner diameter. The reactor temperature was controlled by a digital temperature controller equipped with a K-type thermocouple. The reactor was heated to 800 °C at a heating rate of 15 °C min^−1^ under an ultra-high purity grade nitrogen (N_2_) flow atmosphere (99.999%; Thai-Japan Gas Co., Ltd., Phra Nakhon Si Ayutthaya, Thailand) at 150 mL min^−1^ and then maintained at 800 °C for 30 min. The flow was then switched to anhydrous NH_3_ (99.95%; Thai-Japan Gas Co., Ltd., Thailand) at the same flow rate and the sample was maintained at 800 °C in the reactor for 2 h. After that the reactor was cooled down to 120 °C, the flow switched to N_2_ and cooled down to room temperature. The obtained NOCn sorbent was kept in a dark bottle and stored in a desiccator. The nitrogen-doped carbonaceous sorbent (NCn) was obtained in the same method except substituting Cn for OCn.

### 2.2. Measurement of CO_2_ Adsorption

Static volumetric analyses were performed on a BELSORP-mini II (MicrotracBEL Corp., Nagoya Japan) instrument to evaluate the CO_2_ adsorption capacity of these four types of sorbents (Cn, OCn, NCn and NOCn). For this, 60 mg of sorbent was contained in the glass vessel and placed to the port sample of the instrument. Before the measurement, each sample was degassed under vacuum pressure at 120 °C for 6 h, followed by heating to 150 °C and then maintained at 150 °C for 2 h. The sample was cooled down to 25 °C under vacuum pressure. The reactor was then connected to the water bath to control the temperature of the influent gas, and the operating adsorption at the designed temperature of 25 °C. The measurement of CO_2_ adsorption at the given temperature was then done immediately, routing CO_2_ with 99.99% purity (Chubu Air Water Inc., Nagoya, Japan) to the sample until approaching CO_2_ saturation at various pressures up to 1 bar. When the pressure-change in the adsorption chamber was less than 30 Pa within 300 s, it was taken to mean the adsorption equilibrium was reached and the equilibrium pressure was then recorded.

The NOCn sorbent, which expressed the higher CO_2_ adsorption capacity, and the original Cn sample were then selected to study the effect of temperature on the CO_2_ adsorption capacity. The operating step was the same as above, except the operating temperature was varied (35, 45 and 55 °C). The dead volume of the sample vessel was considered in each measurement. The adsorption data of the NOCn sorbent and the original Cn sorbent were also employed to study thermodynamics. The adsorption isotherms were fitted to two-parameter (Langmuir and Freundlich) and three-parameter (Sips and Toth models) models.

### 2.3. Characterization of the Sorbents

A Rigaku SmartLab diffractometer with monochromatic Cu Kα source (λ = 0.154 nm) at 45 kV, 200 mA, and 0.02° s^−1^ scan speed rate was used to record X-ray diffractograms (XRD) to identify the intrinsic crystallinity of the sorbents. The XRD patterns were recorded over a 2θ range from 5–80°. The magnitude of the interlayer spacing (d_002_) of carbon nanocrystallites was calculated from the peak at the (002) plane using the Bragg equation.

After hand-grinding the sample to a fine powder, the powder was placed over a grid in order to analyze the morphology of the sorbent sample by transmission electron microscopy (TEM) using a JEM-2500SE (JEOL, Tokyo, Japan) microscope under an accelerating voltage of 200 kV. The selected area electron diffraction (SAED) was also performed on this instrument.

Raman spectra were recorded on a Raman Microscope (inVia confocal Raman Microscope, Renishaw Co. Ltd., Tokyo, Japan) with an excitation wavelength of 532 nm over a Raman shift range from 500–2000 cm^−1^ at a resolution of 1.3 cm^−1^. The in-plane (L_a_) crystallite size was calculated based on the D/G peak intensity ratio.

The N_2_ adsorption/desorption isotherm of sorbent(s) was determined via volumetric analysis on a BELSORP mini II analyzer at −196 °C. The Brunauer-Emmett-Teller (BET) equation was used to calculate the specific surface area. The microporosity of samples was investigated using the t-plot calculated from the data series of N_2_ adsorption isotherms. The adsorption measurements were performed using the BELSORP analysis software version 5.3.3.0. Prior to measurement, moisture in the samples was eliminated by drying the sample in a vacuum oven at 100 °C for 12 h. The sample was then degassed under vacuum pressure at 150 °C for 2 h.

To evaluate the surface chemical state of the sorbent(s), X-ray photoelectron spectroscopy (XPS; JPS-9010MC, JEOL) was employed. Monochromatic Mg Kα radiation with 1253.6 eV was used as an excitation source. The emission current and the anode voltage were driven at 25 mA and 10 kV, respectively. The BE was calibrated using the C 1s peak at 284.5 eV for pristine carbon. An 80:20 Gaussian-Lorentzian ratio of line function with the subtraction of the Shirley background was used to fit the curve.

The total amount of nitrogen on the sorbent(s) was examined via a CHN/O elemental analyzer (Perkin Elmer 2400 Series II) with a thermal conductivity detector. The sample (2 mg) on aluminium foil was accurately weighed and then placed in the sample chamber before being combusted in a pure oxygen stream under a static condition. Each sample was analyzed at least three times, and the results are reported in terms of the average of the concordant results.

## 3. Results and Discussion

### 3.1. Characterization of Sorbents

**Figure 2:**
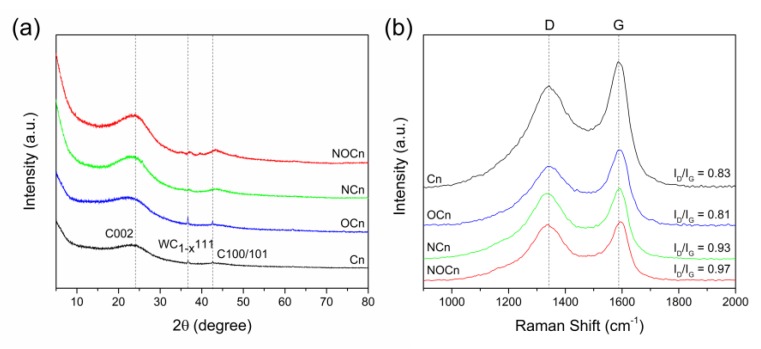
Representative (**a**) XRD patterns and (**b**) Raman spectra of the different adsorbents.

To identify the synthesized solid particles by their intrinsic crystallinity, XRD was performed. The obtained XRD patterns (Figure 2a) revealed two main broad peaks in each profile of carbon nanocrystallite located at a 2θ of around 24° and 43° and one peak of carbide located at a 2θ of around 37°. The peak at a 2θ of 24° corresponded to the (002) plane of the turbostratic carbon [23], while that at 43° was the (100)/(101) plane of carbon, reflecting the hexagonal ring structure of carbon (JCPDS card no.75-1621). The peak at a 2θ of 37° represented the (111) plane of tungsten carbide (WC_1-x_), formed from the sputtering of the tungsten electrodes during the plasma discharge in the SPP [25]. No other peaks were observed in the XRD patterns, inferring no contamination (impurity) in the carbon crystallite. Observing the peak at a 2θ of 24° for the Cn and OCn samples, its maximum was shifted towards a smaller 2θ value in Ocn than in Cn, which indicates that some oxygen was doped on the carbon framework, and also led to an expansion of the interlayer carbon sheet (d_002_) space from 0.370 nm for Cn to 0.383 nm for Ocn (Table 1). After nitriding, the Cn and Ocn samples at 800 °C to obtain NCn and NOCn, respectively, the shift in the maximum 2θ peak of 24° was still found. A change in the interlayer spacing value (d_002_) was also found (Table 1). The magnitude of d_002_ was about 0.375 nm for NCn and 0.372 nm for NOCn. This inferred that the mechanism of nitridation of Cn and Ocn was not the same. However, the d_002_ values of the particles synthesized here are higher than the 0.335 nm interlayer spacing of ideal graphite, reflecting a lower degree of graphitization on the carbon framework. Additionally, after nitriding, the peak at a 2θ of 37° was shifted slightly to a larger 2θ value and became broader. This was because the tungsten carbide might have been reduced by hydrogen gas [26] produced from the decomposition of NH_3_ at the high temperature (800 °C) used in the nitridation.

Representative Raman spectra of the Cn, Ocn, NCn and NOCn samples are shown in Figure 2b. Only two bands were found in the spectra; the G band at around 1585 cm^−1^ and the D band at around 1335 cm^−1^. The G band represents the vibration of sp^2^ orderly bonded carbons, while the D band corresponds to the vibration of sp^3^ carbon atoms, indicating the defects on the graphene layer. The intensity ratio of the D and G bands (I_D_/I_G_) was used to evaluate the degree of defection or disorder on the carbon framework [27,28], with the results summarized in Table 1. The Cn and Ocn samples showed a similar I_D_/I_G_ ratio value of around 0.83 and 0.81, respectively. After nitriding at a high temperature to obtain NCn and NOCn, the I_D_/I_G_ ratio was increased to 0.93 for NCn and 0.97 for NOCn. This indicated that nitrogen doping caused defects on the carbon framework [29], and then the structure became less crystalline. A slight shift towards a higher Raman shift of the G band was found in the spectra of the NCn and NOCn compared to that in the Cn and Ocn, respectively. The inverse of I_D_/I_G_ ratio was applied to calculate the size of the in-plane crystallite, L_a,_ via the Tuinstra-Koenig relationship [30], shown in Equation (1):

(1) $$L_{a}\left(\mathrm{nm}\right)\;={\;C\lambda }^{4}{\left(I_{D}/I_{G}\right)}^{-1},$$

where C is a constant (2.4 × 10^−10^ nm^−3^), and λ is the excitation laser wavelength (532.1 nm). From Table 1, the L_a_ crystallite diameter of Cn was around 23.18 nm, while it was around 23.75 nm for OCn. Thus, an increase in the magnitude of L_a_ was found when doping oxygen on the carbon framework. After nitriding, the crystallite diameter became smaller, being 20.69 nm for NCn and 19.83 nm for NOCn, which might be due to the decomposition of the carbon during nitriding at a high temperature.

**Figure 3:**
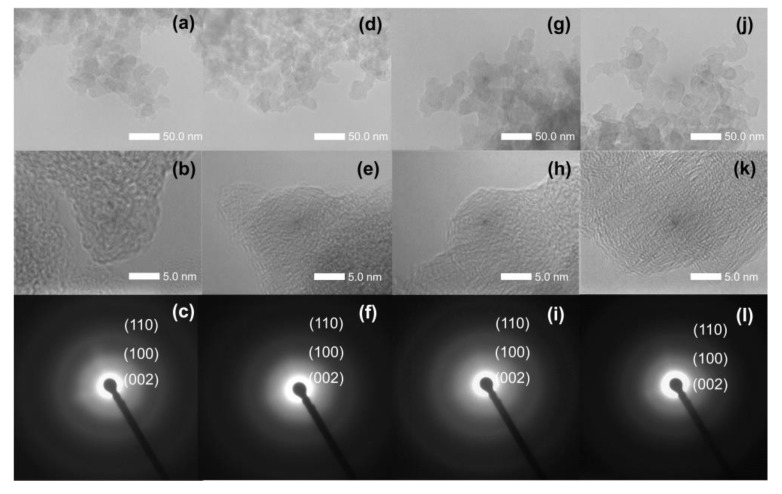
Representative TEM images of (**a**,**b**) Cn, (**d**,**e**) NCn, (**g**,**h**) OCn and (**j**,**k**) NOCn and SAED patterns of (**c**) Cn, (**f**) NCn, (**i**) OCn and (**l**) NOCn.

Representative TEM images of the different types of particles are shown in Figure 3. The size of these particles was less than 50 nm, indicating they are nanoparticles. From the negative bright-field images of the Cn, OCn, NCn, and NOCn samples, the fringes were in a non-directive arrangement. This contributes to the 002 plane of carbon surrounded by an amorphous phase. However, the skeleton carbon in each particle was similar to each other. As can be seen from the SAED images of Cn (Figure 3c), NCn (Figure 3f), OCn (Figure 3i) and NOCn (Figure 3l), the structure of the particles was mainly amorphous with some polycrystalline. Compared to the SAED of the Cn and OCn samples, more blurred bright onion rings were observed in the SAED of the OCn sample, since some oxygen confounded with the carbon framework, while NCn and NOCn showed more blurred bright onion rings than C and OCn, respectively. During nitriding, some nitrogen atoms passed into the carbon framework, while some carbon atoms at the edge reacted with active species of NH_3_ at high temperature. Thus, the crystallinity of the nitrided samples was decreased, and they became more amorphous.

**Figure 4:**
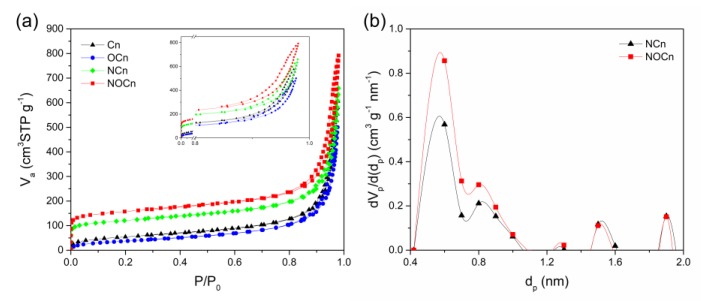
The representative of (**a**) N_2_ adsorption/desorption isotherms at −196 °C and (**b**) Micropore size distribution.

Representative equilibrium N_2_ adsorption/desorption isotherms at −196 °C for the Cn, OCn, NCn and NOCn samples are demonstrated in Figure 4a. At the initial relative pressure, the first and steepest slope was found. The isotherm of NOCn and NCn exhibited a greater slope than the Cn and OCn samples, which indicated the development of ultramicropores with a diameter of less than 0.7 nm, during the nitriding at a high temperature. From the micropore size distribution via the MP-plot, as shown in Figure 4b, it was evident that most pores in NCn and NOCn had a size of around 0.6 nm. Further increasing the relative pressure up to 0.8, N_2_ uptake gradually rose. A higher amount of N_2_ uptake was found when increasing the relative pressure from 0.8 to 1.0. Moreover, an adsorption/desorption hysteresis loop was observed in all isotherms (upper right corner of Figure 4a), which is mainly caused by capillary condensation. Based on IUPAC classifications [31], the isotherms of Cn and OCn expressed the integration of type II and type IV with an H3 hysteresis loop, meaning a non-porous sorbent with interparticle mesopores. The isotherms of NCn and NOCn were attributed to type I and type IV with an H3 hysteresis loop, meaning a porous sorbent with interparticle voids. It is noted that the isotherm of these samples changed from type II to type I after nitriding at 800 °C.

The specific surface area of the samples was calculated using the BET equation (S_BET_) [32], t-plot method (S_t_) [33,34], and the results summarized in Table 2. The S_BET_ of Cn and OCn were closed to the S_t_ obtained from t-plot method. The Cn sample had a higher specific surface area with a smaller average pore size than the OCn sample. However, the S_t_ of NCn and NOCn were higher than their specific surface area obtained from the BET equation, due to a presence of micropores. Differences in the type of working solution used in the SPP leads to a disparity of the synthesized particles. After nitriding, the OCn, the S_t_ of NOCn (679.8 m^2^ g^−1^) was about five-fold higher than that for OCn (137.5 m^2^ g^−1^), while the average pore diameter decreased 2.6-fold from 22.4 nm to 8.6 nm. For the nitridation of Cn to obtain NCn, the St was increased around 2.7-fold from 191.4 m^2^ g^−1^ to 521.3 m^2^ g^−1^, while the average pore diameter size was decreased approximately 2.1-fold from 19.7 nm to 9.3 nm. This reflects the development of micropores during the nitriding at 800 °C. The t-plot method [33,34] was then suggested to determine the micropore area and volume of NCn and NOCn. Note that since both Cn and OCn are non-porous, they could not express any information on micropores. The NOCn showed a two-fold higher micropore area and volume than NCn, and so NOCn should exhibit a higher CO_2_ adsorption capacity.

The surface elemental analyses (Table 3) revealed that the oxygen level at the surface of the sample increased from 4.18 at % for Cn to 7.21 at % for OCn, with a 1.77-fold higher oxygen/carbon (O/C) ratio in OCn than that in Cn. This supported that the oxygen-doped samples were successfully synthesized in the one-step SPP at room temperature and atmospheric pressure. It is noted that the N-content was not observed in the Cn sample or the OCn sample. After nitriding at 800 °C to obtain the NCn and NOCn samples, the nitrogen/carbon (N/C) ratio increased to 0.013 and 0.024 for NCn and NOCn, respectively. The nitrogen content in NOCn was around 1.78-fold higher than in NCn, indicating a different way to react with the active species derived from NH_3_ during nitriding. The NH_3_ that routed to the nitriding process at high temperature was decomposed to active species that then react with the carbon on the surface of Cn to obtain the NCn with various N-bonds, while they replace the oxygen on the surface of OCn [35], and form a bond with the carbon framework to achieve NOCn, as evidenced in the XPS results (Table 4). Even though the N/C ratio after nitriding increased, the O/C ratio became smaller from 0.044 for Cn to 0.017 for NCn and from 0.078 for OCn to 0.025 for NOCn. Moreover, the bulk elemental analyses (Table 3) showed that the NOCn and NCn samples had a lower carbon level than the OCn and Cn samples, respectively. The decreased O/C ratio and carbon level were perhaps, due to the decomposition of oxygen and carbon at the elevated temperature (800 °C) during nitridation. The change in the carbon level might drive the development of the microporosity, as evidenced in the pore structure studies (Table 2).

**Figure 5:**
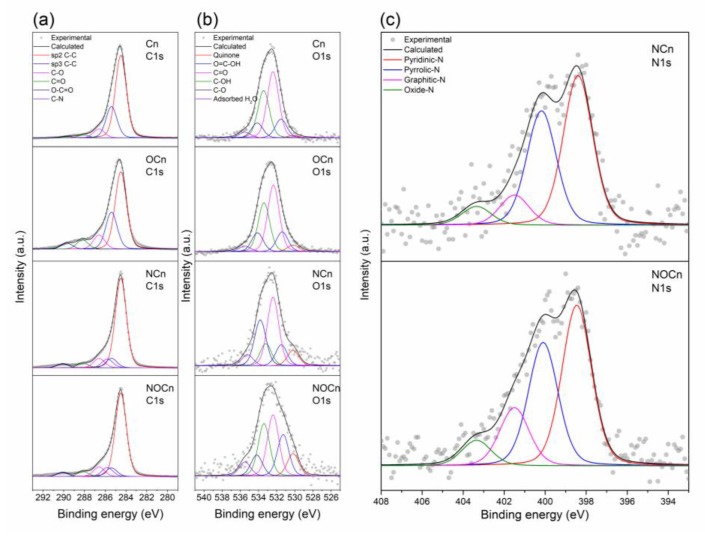
Representative XPS spectra of the Cn, OCn, NCn and NOCn samples for the (**a**) C 1s, (**b**) O 1s and (**c**) N 1s regions of the nitrided samples (NCn and NOCn).

Representative XPS spectra of C 1s, O 1s, and N 1s are shown in Figure 5 and summarized in Table 4. For the C 1s region, the major bands represent the C–C sp^2^ bonding at a BE of 284.5 eV and C–C sp^3^ ordering at a BE of 285.4 eV. This implied that the carbon framework of the samples was composed of ordered and disordered carbon. The long-tail peak in the C 1s region refers to the heteroatom bonds of C–O at 286.5 ± 0.1 eV, C=O at 288.1 ± 0.2 eV and O–C=O at 289.9 ± 0.2 eV. The C–N band at 285.8 eV was found in the spectra of NCn and NOCn, supporting the successful synthesis of NCn and NOCn via nitridation. For the O 1s region, the XPS spectra revealed quinone at 530.2 eV, O=C–OH at 531.4 ± 0.2 eV, C=O at 532.3 ± 0.1 eV, C–OH at 533.3 ± 0.1 eV, C–O at 534.0 ± 0.2 eV and O–H at 535.5 eV. The presence of the O–H bond was due to the moisture adsorbed on the sample. Only the spectra of the nitriding samples expressed the N 1s region, which represented various N-forms on the surface, such as pyridinic at 384 ± 0.1 eV, pyrrolic-N at 400.2 ± 0.1 eV, graphitic-N at 401.3 ± 0.2 eV and pyridinic N-oxide at 403.5 ± 0.2 eV. Most of N-contribution belonged to nitrogen bonding on edge, at around 90.4% for NCn and 84.2% for NOCn, whereas, the nitrogen chemical bond in bulk (graphitic-N) existed at about 9.6% and 15.8%, respectively. Doping nitrogen at the edge was done by replacing the surface oxygen-functional group with the active species of NH_3_ (NH and NH_2_ radicals) [35,36]. There is some difficulty in doping nitrogen atoms on the bulk, due to the requirement to break the strong bonds of the carbon matrix [29]. The major N-contribution in the N 1s region of the NCn and NOCn samples was the pyridinic form, which is attributed to its adsorption performance. Moreover, the W4f band of elemental tungsten at a BE of around 33 eV was not observed in the XPS spectra of all samples, since the amount of contaminated tungsten was quite low.

### 3.2. Performance of CO_2_ Adsorption

The influence of the type of adsorbent and temperature on the CO_2_ adsorption capacity was investigated by volumetric analysis and is reported in terms of mmol CO_2_ adsorbed g^−1^ adsorbent (dry weight).

**Figure 6:**
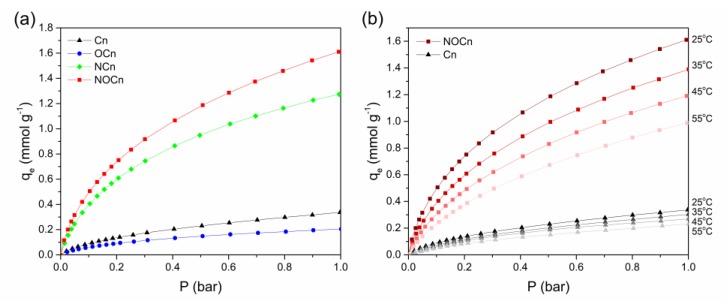
CO_2_ adsorption capacity (**a**) at 25 °C as a function of the pressure and (**b**) as a function of the pressure at various temperatures.

The four types of particles synthesized here (Cn, OCn, NCn, and NOCn) were evaluated for their CO_2_ adsorption capacity at 25 °C (Figure 6a). The capacities of each sample increased with pressure, due to the thermodynamic driving force [37]. The adsorption phenomenon of gaseous CO_2_ molecules on the solid adsorbent could be explained as when the gaseous molecules come close to the adsorbent surface, they induced attractive interactions between them [38,39]. Increasing the equilibrium pressure then led to an increased amount of gas molecules covered on the surface, resulting in higher adsorption capacity. The maximal capacity of each sample was found at 1 bar and was ranked (lowest to highest) as: OCn (0.20 mmol g^−1^) < Cn (0.34 mmol g^−1^) < NCn (1.28 mmol g^−1^) < NOCn (1.63 mmol g^−1^). Based on the textural properties of the sorbent (Table 2), those with a higher specific surface area and smaller average pore diameter size expressed a higher adsorption capacity. Moreover, NOCn had a higher micropore area and volume than NCn, promoting a higher degree of CO_2_ adsorption [40,41]. In addition, the presence of the nitrogen functionality, especially the formation of pyridine-like species, on the surface of the carbon (Table 4) enhanced the CO_2_ adsorption capacity, and so the nitrided samples expressed a higher CO_2_ adsorption capacity than those without nitriding by about 8.15- fold for NOCn and 3.76-fold for NCn, compared to OCn and Cn, respectively. The higher adsorption capacity was effectively achieved by the improved interaction between the acidic CO_2_ molecules and the active nitrogen basic sites on the adsorbent surface [42]. In conclusion, the CO_2_ adsorption capacity depended not only on the textural properties of the adsorbent, and particularly the micropore area, but also on the nitrogen functionality on the surface of the carbonaceous skeleton of the adsorbent. Due to the origin of Cn and the higher adsorption capacity of NOCn, they were selected for further study.

The effect of the temperature on the CO_2_ adsorption capacity of Cn and NOCn at various pressures is shown in Figure 6b (data provided in Table S1). It can be seen clearly that, at the same equilibrium pressure, the CO_2_ adsorption was decreased from 1.63 mmol g^−1^ to 1.37, 1.18 and 0.99 mmol g^−1^ for NOCn, and from 0.34 mmol g^−1^ to 0.32, 0.28 and 0.24 mmol g^−1^ for Cn, as the temperature increased from 25 °C to 35, 45 and 55 °C, respectively. The effect of pressure on the CO_2_ adsorption capacity differed from that of the temperature. The pressure played an important role in the thermodynamic driving force to push the adsorption forward, and so the CO_2_ adsorption capacity increased with increasing pressure (Figure 6a). For the temperature, a decrease in CO_2_ adsorption capacity was observed with increasing temperatures (Figure 6b). Increasing the temperature caused the CO_2_ molecules to diffuse faster. Therefore, fewer molecules were able to interact with the active site of the adsorbent. Moreover, at higher temperatures, the surface CO_2_ molecules were desorbed into the surrounding gas once there was adequate energy to overcome the gas-solid interaction [43]. Therefore, increasing the temperature attenuated the CO_2_ uptake, in agreement with the exothermic nature of the process. The CO_2_ adsorption capacity of NOCn was greater than that of Cn at the same pressure and temperature. This reflects that these adsorbents have different active sites for CO_2_ binding on their surface. It also infers that CO_2_ has stronger interactions with the active sites on NOCn than on Cn, and so more CO_2_ molecules covered the surface of NOCn, leading to a higher CO_2_ adsorption capacity.

### 3.3. Thermodynamic Studies

To gain insight into the adsorption phenomenon and to determine the thermodynamic parameters, the affinity of adsorbate-adsorbent interaction at the equilibrium state was investigated when there was no coverage of adsorbate on the surface of the fresh adsorbent. In principle, at a very low pressure (P → 0), the adsorbate-adsorbent forces are the most dominant, and so we can apply Henry’s law (Henry’s law region). Henry’s constant is directly related to the adsorbate-adsorbent interaction and represents affinity. To obtain Henry’s constant, we used the Virial equation [44], as shown in Equation (2):

(2) $$P/q=1/K_{H}\exp\left(2A_{1}q+3/2A_{2}q^{2}+\ldots \right),$$

where K_H_ is the Henry’s constant (mmol g^−1^ bar^−1^), P is the pressure (bar), q is the amount adsorbed (mmol g^−1^), and A_1_ and A_2_ are the virial coefficients. The virial plot between the natural logarithm function of the ratio of P to q (P/q) and q is then done. After linearization, it gives the straight line approaching the axis (q → 0), the -ln K_H_ is then obtained from the intercept, whereas, the virial coefficients with higher order (A_2_, A_3_, …) could be neglected. The K_H_ at different temperatures of CO_2_ adsorption on Cn and NOCn are shown in Table 5. Over the whole temperature range, higher Henry’s constants for adsorption were found on NOCn, which means that NOCn exhibited a higher affinity for CO_2_ adsorption than Cn. It is noticed that the value of K_H_ became smaller with increasing temperatures, inferring that CO_2_ adsorption on the adsorbent was less favorable at higher temperatures, and so the CO_2_ capacity was decreased in accord with the temperature effect on the capacity, as discussed above and in Section 3.2.

To understand the nature of the adsorption phenomenon and type of adsorption, the other thermodynamic parameters were investigated via (i) van ’t Hoff equation for the enthalpy change of adsorption ($\Delta H_{\mathrm{ads}}^{ο}$.) [45], as shown in Equation (3); (ii) the fundamental Gibb’s free energy equations, for Gibb’s free energy ($\Delta G_{\mathrm{ads}}^{ο}$), as shown in Equation (4), and the entropy change of adsorption ($\Delta S_{\mathrm{ads}}^{ο}$), as shown in Equation (5):

(3) $${d\;\ln\;K}_{H}/d(1/T)=-\Delta H_{\mathrm{ads}}^{ο},$$

(4) $$\Delta G_{\mathrm{ads}}^{ο}=\;-{\mathrm{RTln}\;K}_{H},$$

(5) $$\Delta G_{\mathrm{ads}}^{ο}=\Delta H_{\mathrm{ads}}^{ο}-T\Delta S_{\mathrm{ads}}^{ο}.$$

The assumption is that the enthalpy and entropy changes are essentially constant over the small range of studied temperatures. The difference in each adsorption temperature interval should not exceed 10 K. These thermodynamic parameters can be used as a crucial key to characterizing the adsorption process.

**Figure 7:**
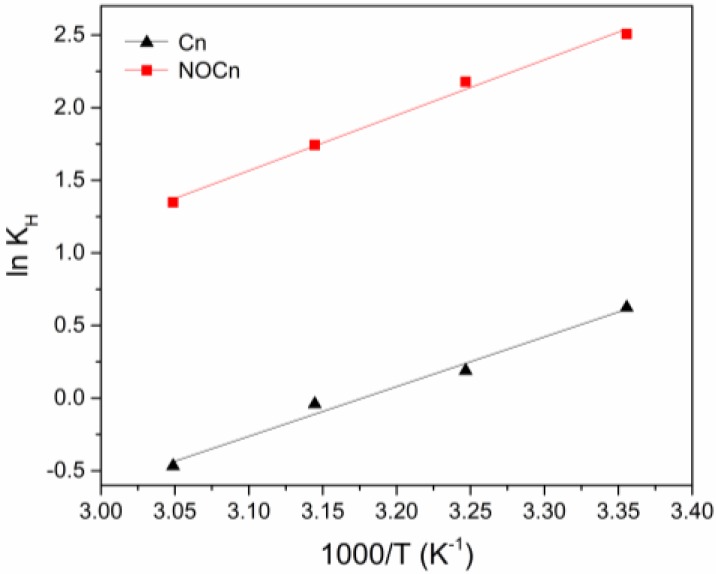
Van ’t Hoff plot of Cn and NOCn.

The van ’t Hoff plot (natural logarithm function of K_H_ as a function of the reciprocal temperature) was a straight line (Figure 7), where the gradient and intercept of the line were $-\Delta H_{\mathrm{ads}}^{ο}$/R and $\Delta S_{\mathrm{ads}}^{ο}$/R, respectively. The thermodynamic parameters were then determined and are shown in Table 5. At 25 °C, Cn and NOCn had negative $\Delta G_{\mathrm{ads}}^{ο}$ values, which means the adsorption of CO_2_ on both sorbents was spontaneous at this temperature. The $\Delta G_{\mathrm{ads}}^{ο}$ values for NOCn at different temperatures were more negative than those for Cn, confirming that the CO_2_ adsorption on NOCn is more thermodynamically feasible. Nitridation at high temperature caused micropore development and doped nitrogen atoms on the surface, which then enhanced the CO_2_ adsorption capacity. When increasing the temperature, the $\Delta G_{\mathrm{ads}}^{ο}$ from NOCn and Cn became less negative, which means that the adsorption had a lower degree of spontaneity at higher temperatures. At lower temperatures, the CO_2_ molecules diffused to the more energetically favorable active sites on the surface of adsorbent to form a surface layer of CO_2_ molecules. Consequently, a higher adsorption capacity was obtained at a lower temperature, as discussed above. At a higher temperature, CO_2_ diffuses faster, and CO_2_ molecules with a weaker interaction with the less energetically favorable active sites on the sorbent surface can then desorb and diffuse back to the gaseous bulk phase.

The sign of $\Delta H_{\mathrm{ads}}^{ο}$ indicates the heat inflow/outflow to the adsorption system, where a negative sign meant that the nature of the adsorption process on Cn and NOCn was exothermic. The total energy released during the formation of a bond between CO_2_ and the active sites on the sorbent was greater than the total energy used in breaking the adsorbed CO_2_ bond. The absolute value of $\Delta H_{\mathrm{ads}}^{ο}$ from NOCn was greater than that from Cn, meaning that a larger amount of heat energy is released to the surrounding after adsorbing CO_2_ molecules on the NOCn surface. This also reflected the stronger nitrogen-active site on NOCn. Moreover, the absolute magnitude of $\Delta H_{\mathrm{ads}}^{ο}$ revealed the type of adsorption, where $\Delta H_{\mathrm{ads}}^{ο}\;\mathrm{is}\;$< 40 kJ mol^−1^ and > 80 kJ mol^−1^ for physisorption and strong chemisorption, respectively, [46]. Thus, the major mechanism of CO_2_ adsorption on Cn and NOCn was physisorption, where the active site on the adsorbent surface attracts CO_2_ through weak Van der Waals forces.

For $\Delta S_{\mathrm{ads}}^{ο}$, the sign corresponded to the degree of randomness of the adsorption process. It was clearly seen (Table 5) that lower randomness of the system was observed during the CO_2_ adsorption onto NOCn than onto the Cn surface. The gaseous CO_2_ in the bulk phase moved randomly, while the CO_2_ adsorbed on the surface could not move freely, due to their interaction force. The stronger interaction force between CO_2_ and the nitrogen-active site on the NOCn caused a more ordered stage with less randomness.

### 3.4. The Selectivity of CO_2_ Adsorption

**Figure 8:**
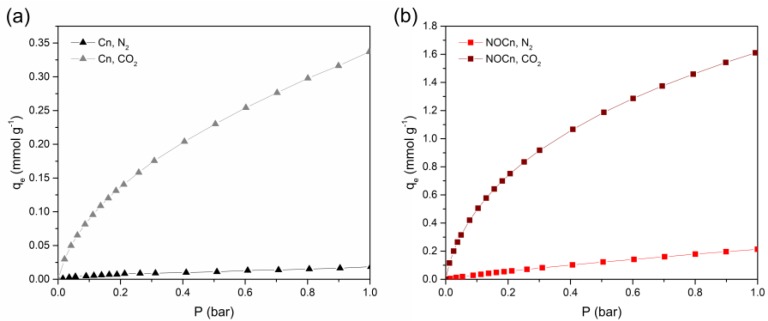
N_2_ and CO_2_ adsorption isotherm at 25 °C on (**a**) Cn and (**b**) NOCn.

A potential sorbent should express a selective adsorption ability. Herein, the CO_2_ and N_2_ adsorption isotherms on Cn and NOCn at 25 °C (Figure 8) were then applied to study the selective CO_2_ adsorption via the affinity of adsorbate-adsorbent interactions in terms of Henry’s constant (as shown in Equation (2)). From Figure 8, it can be seen that Cn and NOCn expressed an N_2_ adsorption capacity of around 0.02 mmol g^−1^ and 0.21 mmol g^−1^, respectively, and CO_2_ adsorption capacity of about 0.34 mmol g^−1^ and 1.63 mmol g^−1^, respectively. The Henry constant for the N_2_ adsorption ($K_{{H,N}_{2}}$) and CO_2_ adsorption ($K_{{H,\mathrm{CO}}_{2}}$) on Cn and NOCn are summarized in Table 6, where the selective CO_2_ adsorption on Cn and NOCn was then exhibited as the ratio of $K_{{H,\mathrm{CO}}_{2}}/K_{{H,N}_{2}}$, and shown in Table 6. $K_{{H,N}_{2}}$ was about 0.11 for Cn and 0.42 for NOCn, while $K_{{H,\mathrm{CO}}_{2}}$ was around 1.86 for Cn and 12.26 for NOCn, respectively. It was noted that the magnitude of $K_{{H,\mathrm{CO}}_{2}}$ for both sorbents was higher than that of $K_{{H,N}_{2}}$. This reflected that both sorbents had a greater affinity to CO_2_ than N_2_. Higher values of $K_{{H,N}_{2}}$ and $K_{{H,\mathrm{CO}}_{2}}$ for NOCn inferred that NOCn had more favorable adsorption. After nitriding to obtain NOCn, the affinity was increased about 6.6-fold or CO_2_ adsorption and 3.8-fold for N_2_ adsorption. This enhanced the CO_2_ adsorption affinity could be because not only micropores were developed during nitridation, but there was also nitrogen doping onto the carbon structure. The ratio of $K_{{H,\mathrm{CO}}_{2}}/K_{{H,N}_{2}}$ was about 16.29 for Cn and 29.22 for NOCn, respectively. This revealed that the sorbents had a selective CO_2_ adsorption ability and the CO_2_ adsorption was more favorable on NOCn than Cn. This is because CO_2_ was attached to the surface of sorbent with stronger induced dipole interactions. Even though CO_2_ and N_2_ are nonpolar molecules, the polarizability and quadrupole moment are 26.5 × 10^−25^ cm^3^ and 4.3 × 10^−26^ esu·cm^2^ for CO_2_ and 17.6 × 10^−25^ cm^3^ and 1.52 × 10^−26^ esu·cm^2^ for N_2_, respectively [47]. Thus, the carbon surface could form stronger interactions at the electron-rich region, particularly the π-system and/or lone pair electron of N atom in the case of NOCn. Therefore, it is more attractive to the position of C^δ+^ of CO_2_.

### 3.5. Cyclic Adsorption/Regeneration

**Figure 9:**
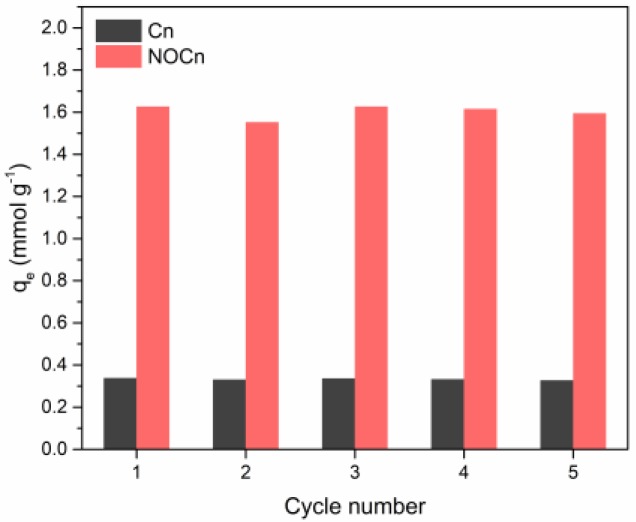
Cyclic adsorption/regeneration performance.

Section 3.4 revealed that the CO_2_ adsorption on Cn and NOCn was physisorption where CO_2_ molecules were weakly attracted by Van der Waals forces. This leads to a high possibility for the regeneration of the sorbent. Regeneration is an essential factor in making a sorbent for economical usage. It reduces resource requirement by regenerating spent sorbent and reuse for adsorption application. To test the regeneration of Cn and NOCn, multi-cycled absorption-desorption cycles were conducted. A fresh sorbent was used to saturation in the adsorption procedure as mentioned. After adsorption at 25 °C was completed, the sorbent was regenerated at a temperature of 130 °C under a low pressure (< 0.1 bar) for 2 h and then followed the same CO_2_ adsorption procedure. The results are shown in Figure 9. The change in the adsorption capacities after five adsorption/regeneration cycles was less than 5% for both Cn and NOCn. This implied that the NOCn and Cn had good stability for multicycle adsorption and evacuation.

### 3.6. Adsorption Isotherm Studies

The adsorption isotherms express a relationship between the amount of CO_2_ adsorbed on the solid adsorbent and the equilibrium pressure when the temperature is kept constant. Only the CO_2_ adsorption on NOCn was studied in this part, due to its higher adsorption capacity. The data were fitted to the two-parameter isotherm models of Langmuir and Freundlich, as shown in Equation (S1) and Equation (S2), respectively. In addition, the data were fitted to the three-parameter Sips and Toth isotherm models, as shown in Equations (S3) and (S4), respectively. These four models are based on different assumptions, which the details were described in supportive information part. To evaluate the goodness of fit the (i) coefficient of determination (R^2^); (ii) Marquardt’s Percent Standard Deviation (MPSD); and (iii) error function based on the normalized standard deviation (%Err) were introduced. The best-fit model was chosen based on the magnitude of R^2^ (close to unity) and minimal values of MPSD and %Err. These equations are shown as Equations. (S6)–(S8). Also, all of the model parameters must be a non-negative value. Each model parameter and the magnitude of the three different tests of fitting the data for NOCn to the isotherm models are shown in Table S2. All models exhibited an R^2^ of more than 0.99 and an MPSD less than 0.15, but the Toth model had the lowest %Err suggesting the better fitting of the data to the Toth model. Thus, the adsorption isotherm on NOCn over the whole pressure range at all temperatures can be best characterized via the Toth isotherm. This suggested that the adsorption occurred on heterogeneous active sites on the sorbent surface with different BEs.

### 3.7. Adsorption Mechanism

**Figure 10:**
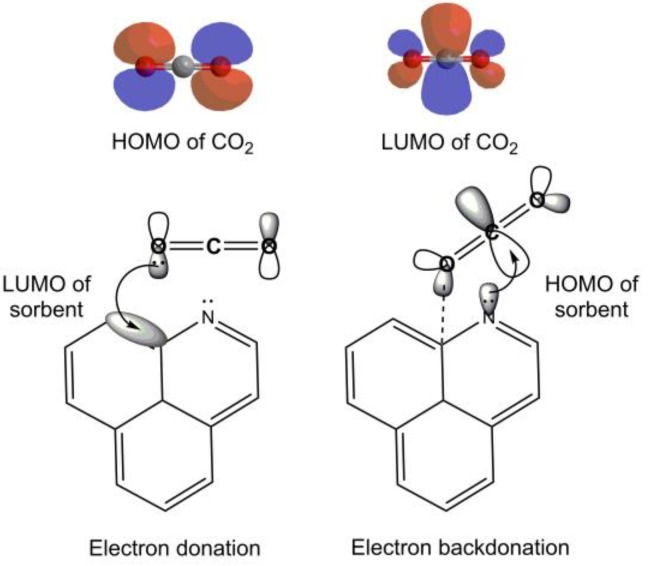
Adsorption mechanism of CO_2_ on the NOCn adsorbent based on electron donation and backdonation.

To explain the mechanism of CO_2_ adsorption on NOCn, the principles of classical chemistry were applied. Nitrogen atoms in the carbon matrix have a greater affinity for CO_2_, since the lone pair electron on the nitrogen atom, especially the pyridinic-N, acts as Lewis base, while the C atom on CO_2_ is an electrophile in nature [20], allowing them to form a Lewis acid-base interaction through N donating an electron to C^δ+^ on the CO_2_ molecule. Since the adsorption of CO_2_ on NOCn was mainly physisorption through weak forces, there was no change in the electronic properties of the CO_2_-adsorbent complex and no significant change in the molecular orbital (MO) level [48]. From the perspective of quantum chemistry, electron donation and electron backdonation, based on the highest occupied MO (HOMO) of the sorbent’s surface interact attractively with the lowest unoccupied MO (LUMO) of the sorbate molecule. The HOMO and LUMO of the CO_2_ molecule and the interaction between the nitrogen atom on the carbon structure and the C atom on CO_2_ are shown in Figure 10. For electron donation, the electron pair in the non-bonding MO (1π_g_), which is located on the oxygen atom of the CO_2_ molecule, was donated to the LUMO on the C site of NOCn. The electrons in the HOMO of the nitrogen atom of NOCn were then back donated to the LUMO of CO_2_ (2π_u_), which in this MO is mainly a 2p_xC_ [49]. In the case of Cn, the pristine carbon without a nitrogen atom had a higher adsorption barrier, compared to NOCn. Therefore, the larger energy gap between the HOMO and LUMO of Cn and the CO_2_ adsorbate was exhibited, leading to a lower adsorption capacity as described above. This also leads to less favorable adsorption, which is in good agreement with the thermodynamic studies. The Cn had a lower Henry’s constant than NOCn, meaning that Cn had a lower CO_2_ adsorption affinity. When doping nitrogen, the energy gap of the electron transfer between CO_2_ and the adsorption site was reduced, which induced the local density of state below the Fermi level [21,50].

## 4. Conclusions

Increasing the O/C ratio on the surface of the carbon framework by 1.77-fold was achieved in one step at room temperature and atmospheric pressure via SPP when substituting a mixture solution of pure benzene and MWF for pure benzene. After nitriding at 800 °C, the nitrogen content in the obtained NOCn was 1.78-fold higher than in NCn, while the degree of graphitization of the carbon framework was decreased and some micropores had developed. The CO_2_ adsorption capacity of NOCn was higher than the other adsorbents (OCn, Cn and NCn). The capacity depended not only on the textural properties of the adsorbent, particularly the micropore area, but also on the nitrogen functionality on the surface of the carbonaceous skeleton. The CO_2_ adsorption capacity increased with increasing pressure, due to the thermodynamic driving force to push the adsorption forward, whereas, increasing the temperature attenuated the CO_2_ uptake, in agreement with the exothermic nature of the process. Thermodynamic studies revealed that the dominant mechanism of CO_2_ adsorption on Cn and NOCn was physisorption and was spontaneous. A higher degree of heterogeneity and stronger bonding forces between CO_2_ and NOCn was revealed. Overall, NOCn showed higher selective CO_2_ adsorption and was the best candidate for multicycle adsorption. The adsorption behavior on NOCn fitted well to Toth’s isotherm, inferring that the CO_2_ adsorption was a monolayer on a heterogeneous surface.

## Figures and Tables

**Table 1:** Structural properties obtained from the XRD and Raman spectroscopy analyses.

Sample	XRD	Raman Spectroscopy	
	d_002_ (nm)	I_D_/I_G_	L_a_ (nm)
Cn	0.370	0.83	23.18
Ocn	0.383	0.81	23.75
NCn	0.375	0.93	20.69
NOCn	0.372	0.97	19.83

**Table 2:** Pore structure parameters of the samples.

Sample	BET		t-Plot		
	Specific Surface Area	Average Pore Size	Specific Surface Area	MicroporeArea	Micropore Volume
	(m^2^ g^−1^)	(nm)	(m^2^ g^−1^)	(m^2^ g^−1^)	(cm^3^ g^−1^)
Cn	190.8	19.7	191.4	-	-
OCn	138.4	22.4	137.5	-	-
NCn	437.8	9.3	521.3	262.7	0.06
NOCn	569.7	8.6	679.8	469.7	0.15

**Table 3:** Elemental composition from the bulk and surface elemental analyses.

Sample	Bulk Elemental Analysis, EA (wt %)			Surface Elemental Analysis, XPS (at %)				
	C	H	N	C	N	O	O/C Ratio	N/C Ratio
Cn	92.99	1.37	-	95.82	-	4.18	0.044	-
OCn	88.13	2.76	-	92.79	-	7.21	0.078	-
NCn	89.21	0.99	1.77	97.05	1.29	1.66	0.017	0.013
NOCn	82.35	1.50	2.76	95.35	2.29	2.36	0.025	0.024

**Table 4:** Deconvolution results of the XPS spectra at C 1s, O 1s and N 1s for synthesized samples.

Region	Bonding	Position (eV)	Relative Percentage (%)			
			Cn	OCn	NCn	NOCn
C 1s	C–C sp^2^	284.5	65.0	53.7	71.5	69.7
	C–C sp^3^	285.4	24.7	25.8	7.5	7.2
	C–N	285.8	-	-	7.0	7.5
	C–O	286.4–286.6	7.0	9.9	7.5	8.1
	C=O	287.9–288.3	2.3	6.5	3.4	4.2
	O–C=O	289.7–290.1	1.0	4.2	3.1	3.4
O 1s	Quinone	530.2	1.5	4.0	8.4	10.5
	O=C–OH	531.2–531.6	12.1	11.9	11.4	19.5
	C=O	532.2–532.4	42.6	40.0	37.2	28.8
	C–OH	533.2–533.4	30.6	29.4	12.1	24.7
	C–O	533.8–534.2	9.6	11.4	24.9	9.9
	Water adsorbed	535	3.6	3.3	6.1	6.6
N 1s	Pyridinic-N	398.4–398.6	-	-	47.8	43.7
	Pyrrolic-N	400.1–400.3	-	-	36.6	33.6
	Graphitic-N	401.1–401.5	-	-	9.6	15.8
	Pyridinic N-oxide	403.3–403.7	-	-	6.0	6.9

**Table 5:** Thermodynamic parameters of CO_2_ adsorption on Cn and NOCn at different temperatures.

Adsorption Temperature (°C)	Cn					NOCn				
	ln K_H_	K_H_^1^	$\Delta {G_{\mathrm{ads}}^{\text{o}}}^{2}$	$\Delta {H_{\mathrm{ads}}^{\text{o}}}^{2}$	$\Delta {S_{\mathrm{ads}}^{\text{o}}}^{3}$	ln K_H_	K_H_^1^	$\Delta {G_{\mathrm{ads}}^{\text{o}}}^{2}$	$\Delta {H_{\mathrm{ads}}^{\text{o}}}^{2}$	$\Delta {S_{\mathrm{ads}}^{\text{o}}}^{3}$
25	0.62	1.86	−1.52	−28.45	−90.39	2.51	12.26	−6.30	−31.73	−85.36
35	0.19	1.21	−0.61			2.18	8.82	−5.44		
45	−0.04	0.96	0.29			1.74	5.71	−4.59		
55	−0.47	0.63	1.20			1.35	3.85	−3.74		

^1^ in unit mmol g^−^^1^ bar^−1^; ^2^ in unit kJ mol^−1^; ^3^ in unit J mol^−1^ K^−1^

**Table 6:** Henry constants on N_2_ and CO_2_ adsorption and selectivity.

Sample	$K_{{H,\mathrm{CO}}_{2}}$	$K_{{H,N}_{2}}$	Selectivity
			$K_{{H,\mathrm{CO}}_{2}}/K_{{H,N}_{2}}$
Cn	1.86	0.11	16.29
NOCn	12.26	0.42	29.22

## Supplementary Materials

The following are available online at https://www.mdpi.com/2079-4991/9/12/1776/s1, Table S1: Tabulated adsorption isotherm data at different temperatures for Cn and NOCn, Table S2: Parameters of fitting the CO_2_ adsorption on NOCn at 25 °C data to different isotherm models.
