# Supplementary material for: Nitriding an Oxygen-Doped Nanocarbonaceous Sorbent Synthesized via Solution Plasma Process for Improving CO2 Adsorption Capacity
Source: Nanomaterials (Basel). 2019 Dec 13;9(12):1776. doi: 10.3390/nano9121776 (PMC6956079; doi:10.3390/nano9121776)
Supplement: Supplementary file 1 [file nanomaterials-09-01776-s001.pdf]

Supporting Information

Nitriding an Oxygen-Doped Nanocarbonaceous Sorbent Synthesized via Solution Plasma Process for Improving CO_2 Adsorption Capacity

Phuwadej Pornaroontham [1], Gasidit Panomsuwan [2], Sangwoo Chae [3], Nagahiro Saito [3], Nutthavich Thouchprasitchai [1], Yuththaphan Phongboonchoo [1,4] and Sangobtip Pongstabodee [1,4,*]

[1] Department of Chemical Technology, Faculty of Science, Chulalongkorn University, 254 Phayathai Road, Pathumwan, Bangkok 10330, Thailand; phuwadej.p@gmail.com (P.P.); nutthavich_t@hotmail.com (N.T.); yuththaphan.p@gmail.com (Y.P.)

[2] Department of Materials Engineering, Faculty of Engineering, Kasetsart University, Bangkok 10900, Thailand; fenggdp@ku.ac.th (G.P.)

[3] Department of Chemical Systems Engineering, Graduate School of Engineering, Nagoya University, Furo-cho, Chikusa-ku, Nagoya 464-8603, Japan; chae@sp.material.nagoya-u.ac.jp (S.C.); hiro@sp.material.nagoya-u.ac.jp (N.S.)

[4] Center of Excellence on Petrochemical and Materials Technology, Chulalongkorn University, 254 Phayathai Road, Pathumwan, Bangkok 10330, Thailand

* Correspondence: sangobtip.p@chula.ac.th (S.P.); Tel.: +66-2-218-7676; Fax: +66-2-255-5831

Table S1. Tabulated adsorption isotherm data at different temperatures for Cn and NOCn.

25 °C		35 °C		45 °C		45 °C	
P bar	q_e mmol g^{-1}	P bar	q_e mmol g^{-1}	P bar	q_e mmol g^{-1}	P bar	q_e mmol g^{-1}
Cn							
0.000	0.000	0.000	0.000	0.000	0.000	0.000	0.000
0.024	0.043	0.023	0.032	0.024	0.023	0.022	0.017
0.046	0.070	0.044	0.053	0.045	0.039	0.042	0.030
0.067	0.090	0.064	0.071	0.065	0.053	0.062	0.042
0.092	0.110	0.089	0.088	0.089	0.067	0.086	0.055
0.117	0.129	0.113	0.105	0.114	0.080	0.112	0.067
0.142	0.145	0.138	0.120	0.139	0.093	0.136	0.078
0.167	0.159	0.163	0.134	0.164	0.105	0.161	0.087
0.192	0.172	0.188	0.147	0.188	0.116	0.186	0.096
0.216	0.185	0.212	0.159	0.213	0.124	0.210	0.106
0.264	0.206	0.261	0.179	0.261	0.142	0.259	0.120
0.313	0.225	0.310	0.198	0.311	0.159	0.309	0.133
0.409	0.258	0.407	0.228	0.408	0.187	0.405	0.159
0.509	0.286	0.506	0.257	0.506	0.213	0.505	0.179
0.608	0.313	0.606	0.280	0.606	0.236	0.603	0.200
0.707	0.337	0.703	0.304	0.704	0.259	0.703	0.216
0.806	0.361	0.803	0.326	0.804	0.278	0.801	0.233
0.905	0.382	0.902	0.346	0.902	0.297	0.901	0.247
1.004	0.402	1.001	0.364	1.001	0.316	0.997	0.266
NOCn							
0.000	0.000	0.000	0.000	0.000	0.000	0.000	0.000
0.012	0.115	0.014	0.095	0.016	0.077	0.019	0.062
0.026	0.200	0.028	0.159	0.030	0.124	0.032	0.096
0.038	0.264	0.040	0.206	0.045	0.169	0.050	0.139
0.049	0.315	0.054	0.256	0.060	0.211	0.082	0.200
0.077	0.420	0.084	0.345	0.083	0.265	0.109	0.246
0.104	0.505	0.107	0.404	0.108	0.318	0.135	0.285
0.130	0.577	0.132	0.462	0.133	0.367	0.160	0.322
0.156	0.642	0.157	0.515	0.158	0.412	0.185	0.356
0.181	0.699	0.183	0.562	0.183	0.454	0.210	0.388
0.207	0.751	0.207	0.608	0.208	0.493	0.256	0.442
0.252	0.835	0.253	0.683	0.254	0.558	0.305	0.496
0.301	0.917	0.307	0.759	0.304	0.620	0.405	0.588
0.408	1.066	0.408	0.888	0.408	0.738	0.504	0.673
0.507	1.187	0.507	0.996	0.502	0.831	0.603	0.747
0.601	1.286	0.606	1.088	0.601	0.917	0.705	0.817
0.695	1.374	0.699	1.169	0.700	0.996	0.801	0.879
0.794	1.459	0.803	1.252	0.796	1.063	0.897	0.934
0.898	1.542	0.895	1.315	0.899	1.132	0.996	0.988
0.993	1.611	1.001	1.390	0.994	1.191		

Adsorption isotherm modelling

Two-parameter isotherm

Lungmuir isotherm $$q_e = q_{l,max}K_lP/1 + K_lP \quad \text{(S1)}$$

Freundlich isotherm $$q_e = K_fP^{n_f} \quad \text{(S2)}$$

where $q_{l,max}$ is the maximum adsorption capacity of a monolayer, K_l is the affinity related to the interactions between the binding site of the adsorbent and adsorbate molecule, K_f is a constant related to the affinity of the sorption, n_f is a heterogeneity factor, which is associated with an exponential energy distribution of interactions between sorption site and sorbate molecule.

Three-parameter isotherm

Sips isotherm $$q_e = q_{s,max}(K_sP)^{n_s}/1 + (K_sP)^{n_s} \quad \text{(S3)}$$

Toth isotherm $$q_e = \chi_t^{1/n_t}q_{t,mc}K_tP/[1 + (K_tP)^{n_t}]^{1/n_t} \quad \text{(S4)}$$

and $$\chi_t = 1/(K_tP_m)^{n_t} + 1 \quad \text{(S5)}$$

where K_s is the adsorption affinity related to the equilibrium constant, n_s is the Sips exponent or heterogeneity factor with a value between 0 to 1, $q_{s,max}$ is the maximal capacity, χ_t is the correction factor to eliminate any overestimation in the saturated adsorption capacity [1-4], K_t is the adsorption affinity, P_m is the equilibrium pressure, when the adsorbent surface is saturated by coverage of adsorbate, $q_{t,mc}$ is the corrected monolayer capacity (mmol g^{-1}) and is based on the specific surface area of adsorbent and n_t is the heterogeneous factor that ranged from 0 to 1. It is noted that if n_s and n_t are equal to 1, then these equations reduce to the Langmuir equation.

For the two-parameter models, the Langmuir equation is derived from the balance of the adsorption rate and desorption rate at a dynamic equilibrium state [5]. It describes monolayer adsorption on a homogenous surface with a constant enthalpy of adsorption. The adsorption is proportional to the surface coverage of adsorbate and is saturated at full coverage. However, in the Freundlich isotherm, multilayer adsorption occurs on a heterogeneous surface, where stronger binding sites are occupied first [6-8], and so saturation adsorption is not expressed in this isotherm [9]. Thus, the adsorption capacity tends to increase as the pressure increases towards infinite.

For the three-parameter models, Sips isotherm explains the behavior of heterogeneous adsorption and is based on a combination of the Langmuir and Freundlich isotherms [7,10]. The equation is reduced to the Freundlich isotherm at infinite dilution and represents a Langmuir characteristic at high pressure. The energy distribution of the binding sites resembles a Gaussian distribution. However, Sips isotherm does not obey Henry's law at a low pressure to infinite dilution [9]. For the Toth isotherm equation, a quasi-Gaussian distribution is applied to overcome the limitations of the Sip's isotherm at low pressure [11], and the equation can describe the adsorption isotherm at either a low or high pressure.

To model the adsorption isotherm, the experimental data were fitted to each model without transformation into a linear form. Nonlinear regression analysis was employed in order to avoid any association with a transformation-bias. To verify the consistency of fit with the data, the (i) coefficient of determination (R^2), (ii) Marquardt's Percent Standard Deviation (MPSD), and (iii) error function based on the normalized standard deviation (%Err) were used. The best-fit model should have the highest value of R^2 (close to 1) and the lowest values of MPSD and %Err. These equations are shown as Eqs. (S6)–(S8) below:

$$R^2 = 1 - \left[\sum_{i=1}^{n}(q_{e,exp} - q_{e,calc})^2 \Big/ \sum_{i=1}^{n}(q_{e,exp} - q_{m,exp})^2\right] \quad \text{(S6)}$$

$$\mathrm{MPSD} = \left(1/n - p\sum_{i=1}^{n}\left[\left(q_{e,exp} - q_{e,calc}\right)/q_{e,exp}{}^{2}\right]\right)^{1/2} \tag{S7}$$

$$\mathrm{Err}(\%) = \left(\sum_{i=1}^{n}\left[\left(q_{e,exp} - q_{e,calc}\right)/q_{e,exp}\right]^{2}/n - 1\right)^{1/2} \times 100 \tag{S8}$$

where $q_{e,exp}$ is the equilibrium capacity from the experimental data, $q_{m,exp}$ is the average experimental equilibrium capacity and $q_{e,calc}$ is the equilibrium capacity calculated from the isotherm model (all in mmol g^{-1}), n is the number of data used and p is the number of parameters of the isotherm (two for the Langmuir and Freundlich models and three for the Sips and Toth models).

Table S2. Parameters of fitting the CO_2 adsorption on NOCn at 25 °C data to different isotherm models.

Isotherm models		Fitting parameters	
Two-parameter isotherm	Langmuir	K_l	2.770
		$q_{l,max}$	2.109
		R^2	0.9933
		MPSD	0.149
		%Err	14.5
	Freundlich	K_f	1.656
		n_f	0.520
		R^2	0.9971
		MPSD	0.132
		%Err	12.9
Three-parameters isotherm	Sips	K_s	0.638
		n_s	0.695
		$q_{s,max}$	3.803
		R^2	0.9999
		MPSD	0.025
		%Err	2.4
	Toth	K_t	2.629
		n_t	0.322
		$q_{t,mc}$	3.96
		χ	1.297
		P_m (bar)	16.4
		R^2	0.9999
		MPSD	0.0052
		%Err	0.49

References

1. Garnier, C.; Finqueneisel, G.; Zimny, T.; Pokryszka, Z.; Lafortune, S.; Defossez, P.D.C.; Gaucher, E.C. Selection of coals of different maturities for CO2 Storage by modelling of CH4 and CO2 adsorption isotherms. *International Journal of Coal Geology* **2011**, *87*, 80-86, doi:10.1016/j.coal.2011.05.001.
2. McClellan, A.L.; Harnsberger, H.F. Cross-sectional areas of molecules adsorbed on solid surfaces. *Journal of Colloid and Interface Science* **1967**, *23*, 577-599, doi:10.1016/0021-9797(67)90204-4.
3. Tóth, J. Some Consequences of the Application of Incorrect Gas/Solid Adsorption Isotherm Equations. *Journal of Colloid and Interface Science* **1997**, *185*, 228-235, doi:10.1006/jcis.1996.4562.
4. Tóth, J. Modifications in Classic Relationships Corresponding to Gas/Solid Physical Adsorption. *Journal of Colloid and Interface Science* **1997**, *191*, 449-455, doi:10.1006/jcis.1997.4973.
5. Langmuir, I. The Adsorption of Gases on Plane Surfaces of Glass, Mica and Platinum. *Journal of the American Chemical Society* **1918**, *40*, 1361-1403, doi:10.1021/ja02242a004.
6. Freundlich, H. Over the Adsorption in Solution. *The Journal of Physical Chemistry* **1906**, *57*, 385-470.
7. Sips, R. On the Structure of a Catalyst Surface. *The Journal of Chemical Physics* **1948**, *16*, 490-495, doi:10.1063/1.1746922.
8. Skopp, J. Derivation of the Freundlich Adsorption Isotherm from Kinetics. *Journal of Chemical Education* **2009**, *86*, 1341, doi:10.1021/ed086p1341.
9. Saadi, R.; Saadi, Z.; Fazaeli, R.; Fard, N.E. Monolayer and multilayer adsorption isotherm models for sorption from aqueous media. *Korean Journal of Chemical Engineering* **2015**, *32*, 787-799, doi:10.1007/s11814-015-0053-7.
10. Sips, R. On the Structure of a Catalyst Surface II. *The Journal of Chemical Physics* **1950**, *18*, 1024-1026, doi:10.1063/1.1747848.
11. Tóth, J. Uniform interpretation of gas/solid adsorption. *Advances in Colloid and Interface Science* **1995**, *55*, 1-239, doi:10.1016/0001-8686(94)00226-3.
